# Comprehensive Analysis of the Transcriptome-Wide m6A Methylome in Pterygium by MeRIP Sequencing

**DOI:** 10.3389/fcell.2021.670528

**Published:** 2021-06-25

**Authors:** Yaping Jiang, Xin Zhang, Xiaoyan Zhang, Kun Zhao, Jing Zhang, Chuanxi Yang, Yihui Chen

**Affiliations:** ^1^Department of Ophthalmology, Yangpu Hospital, Tongji University School of Medicine, Shanghai, China; ^2^Department of Ophthalmology, Huashan Hospital, Fudan University, Shanghai, China; ^3^Department of Cardiology, The First Affiliated Hospital of Nanjing Medical University, Nanjing, China; ^4^Department of Cardiology, Yangpu Hospital, Tongji University School of Medicine, Shanghai, China

**Keywords:** pterygium, m6A, MeRIP sequencing, methyltransferase 3, Hippo signaling pathway, GEO

## Abstract

**Aim:**

Pterygium is a common ocular surface disease, which is affected by a variety of factors. Invasion of the cornea can cause severe vision loss. N6-methyladenosine (m6A) is a common post-transcriptional modification of eukaryotic mRNA, which can regulate mRNA splicing, stability, nuclear transport, and translation. To our best knowledge, there is no current research on the mechanism of m6A in pterygium.

**Methods:**

We obtained 24 pterygium tissues and 24 conjunctival tissues from each of 24 pterygium patients recruited from Shanghai Yangpu Hospital, and the level of m6A modification was detected using an m6A RNA Methylation Quantification Kit. Expression and location of *METTL3*, a key m6A methyltransferase, were identified by immunostaining. Then we used m6A-modified RNA immunoprecipitation sequencing (MeRIP-seq), RNA sequencing (RNA-seq), and bioinformatics analyses to compare the differential expression of m6A methylation in pterygium and normal conjunctival tissue.

**Results:**

We identified 2,949 dysregulated m6A peaks in pterygium tissue, of which 2,145 were significantly upregulated and 804 were significantly downregulated. The altered m6A peak of genes were found to play a key role in the Hippo signaling pathway and endocytosis. Joint analyses of MeRIP-seq and RNA-seq data identified 72 hypermethylated m6A peaks and 15 hypomethylated m6A peaks in mRNA. After analyzing the differentially methylated m6A peaks and synchronously differentially expressed genes, we searched the Gene Expression Omnibus database and identified five genes related to the development of pterygium (*DSP*, *MXRA5*, *ARHGAP35*, *TMEM43*, and *OLFML2A*).

**Conclusion:**

Our research shows that m6A modification plays an important role in the development of pterygium and can be used as a potential new target for the treatment of pterygium in the future.

## Highlights

- Both level of m6A and expression of METTL3 were decreased in pterygium.
- 2,145 Upregulated and 804 downregulated m6A peaks were found in pterygium.
- M6A-targeted genes were enriched in Hippo and Notch signaling pathway.
- 72 Hypermethylated and 15 hypomethylated m6A peaks were differently in mRNA.
- Five hub genes were most related to the development of pterygium.

## Introduction

Post-transcriptional modification plays an important regulatory role in various physiological processes and in disease progression (He et al., 2019). To date, more than 100 RNA modifications have been confirmed (Boccaletto et al., 2018). Among them, N6-methyladenosine (m6A), the result of methylation at the sixth nitrogen of adenine, is the most abundant mRNA modification. m6A has an RRACH consensus motif (wherein each R represents A or G, and H represents A, C, or U) (Zhang et al., 2020a), and its related proteins include “Writer” (*METTL3*, *METTL14*, and *WTAP*), “Eraser” (*FTO* and *ALKBH5*), and “Reader” (*YTHDF1*, *2*, and *3*; *YTHDC1-2*; *IGF2BPs*) (Hu et al., 2020). During the transcription of DNA into RNA, adenosine is modified by the action of the writer proteins *METTL3*, *METTL14*, and *WTAP* at the sixth N position (Schöller et al., 2018), and these m6A methylated bases can be recognized by the reader proteins recognition by the YTH Family Proteins (including *YTHDF1, 2, 3*; *YTHDC1, 2*), which are involved in downstream translation (Liu et al., 2020), mRNA translation (Wang et al., 2015) and degradation (Lee et al., 2020), and accelerated mRNA exit from the nucleus (Roundtree et al., 2017). In addition, bases that have undergone m6A modification are de-methylated in the presence of two enzymes, *FTO* and *ALKBH5*, making the m6A reaction reversible (Chen et al., 2019). Recent studies have shown that m6A is closely involved in many biological functions, such as the regulation of the Epithelial-to-mesenchymal transition (EMT) process, the DNA damage response, gene translation, autophagy, adipogenesis, and the determination of stem cell fate (Zhou et al., 2020).

Pterygium is a triangular fibrovascular tissue that grows from the bulbar conjunctiva of the palpebral fissure to the cornea, and it often occurs on the side of the nose (Chen et al., 2014). According to the latest epidemiological survey, the prevalence of pterygium in Asia is 7% (Ang et al., 2012; Chen et al., 2015; Khanna et al., 2020), while in Africa and South America, it is higher (Nemesure et al., 2008; Fernandes et al., 2020). Therefore, it is necessary to explore the specific mechanism of pterygium. At present, no such research has been published. The literature supports the important role of EMT and DNA damage in the pterygium mechanism (Cimpean et al., 2013; Meshkani et al., 2019). m6A is involved in the regulation of the EMT process and DNA damage response and other biological functions, which suggests that it may be involved in the mechanism of pterygium development. Therefore, we investigated whether there is a connection between m6A and the occurrence of pterygium.

To this end, we used high-throughput sequencing to perform a genome-wide analysis of m6A-tagged transcripts. Through m6A-modified RNA immunoprecipitation sequencing (MeRIP-seq) and RNA sequencing (RNA-seq), we identified differentially methylated peaks and differentially expressed genes in mRNA. Further analyses determined that five genes might be associated with pterygium.

## Materials and Methods

### Sample Collection

The study was approved by the Ethics Committee of Yangpu Hospital, Tongji University and was conducted in accordance with the principles of the Declaration of Helsinki. The severity of pterygium is graded by YC and YJ based on slit lamp examination and slit lamp photography.

The severity of pterygium is graded according to the degree of extension and is divided into four grades as follows (Chien et al., 2013):

Grade 1: The pterygium is still involved in the conjunctiva.

Grade 2: Pterygium involvement includes only the corneal limbus.

Grade 3: Pterygium involvement between the corneal limbus and the pupillary limbus.

Grade 4: Pterygium involvement extends centrally to the pupillary margin.

**FIGURE 1:**
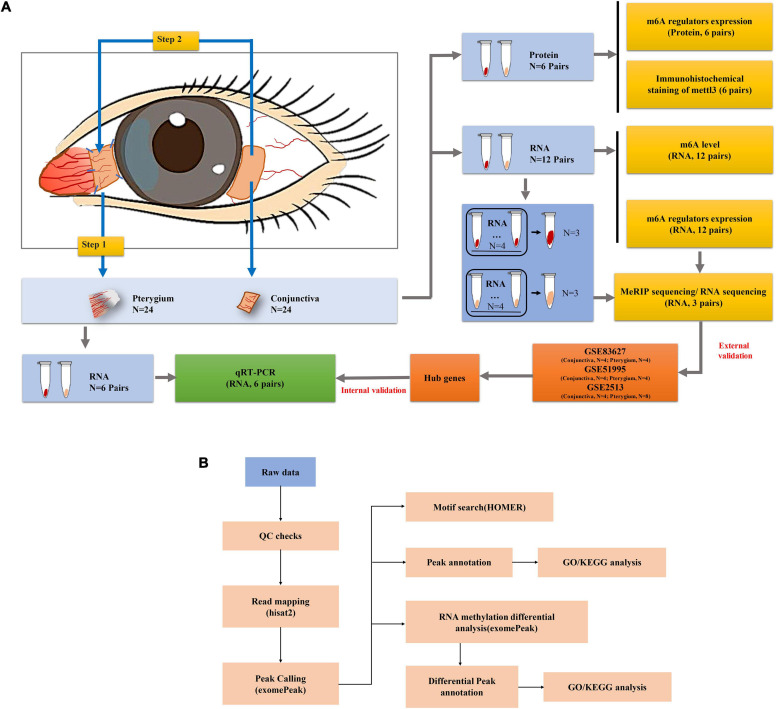
Workflow of the study. **(A)** Workflow of the experimental process. **(B)** Workflow of the data analysis.

A total of 24 patients with pterygium diagnosed as grade III or above were enrolled and they all provided informed consent. People with ocular diseases other than pterygium and those who had undergone ophthalmic surgery were excluded from the study and all patients underwent pterygium excision combined with autologous conjunctival transplantation. Pterygium tissue was obtained by surgical excision, and a small rectangular piece of normal conjunctival tissue was taken from the autograft at the contralateral corneal limbus of the same eye as a control. The collected sample is transferred to a 1.5 mL tube and stored at −80°C for subsequent analysis. Detailed steps are shown in Figure 1A. Clinical information on gender, age, pterygium size, and whether UV exposure were included in the 24 pterygium patients is summarized in Supplementary Table 1.

### Western Blot Analysis

Six pairs of pterygium and conjunctival tissues were lysed for 1 min in cold RIPA buffer [P0013C, Beyotime, the main components are 50 mM Tris (pH 7.4), 150 mM NaCl, 1% NP-40, 0.5% sodium deoxycholate, 0.1% SDS] supplemented with 1 mM PMSF (ST505, Beyotime) using the GeneReady Standard (BSH-2, Life Real). The cells were scraped off and collected into 1.5 mL EP tubes, centrifuged at 12,000 rpm for 15 min and the supernatant was removed for protein quantification using the BCA Protein Concentration Assay Kit (P0012, Beyotime). After denaturation, 30 mg total protein was separated on 10% SDS-PAGE gels and transferred to PVDF membranes. The gels were blocked for 1 h using 5% bovine serum albumin and then incubated overnight at 4°C in the following primary antibodies: anti-*METTL3* (Abcam, 1:1000, cat. No ab195352) (Pan et al., 2021), anti-*METTL14* (Abcam, 1:1000, cat. No ab220030) (Yi et al., 2020), anti-*WTAP* (Abcam, 1:1000, cat. No ab195380) (Chen S. et al., 2020), anti-*FTO* (Abcam, 1:5000, cat. No ab126605) (Baquero-Perez et al., 2019), anti-*ALKBH5* (Abcam, 1:1000, cat. No ab195377) (Zhu et al., 2020) [with *GAPDH* (Proteintech, 1:2000, cat. No 60004-1-Ig) (Lin et al., 2019) as a loading control]. After three washes with TBS-Tween, the proteins were incubated with the corresponding secondary antibodies conjugated to horseradish peroxidase and then detected via enhanced chemiluminescence (ECL) using the iBright CL1000 device (Invitrogen).

### RNA Isolation and Quantitative Real-Time PCR

Using TRIzol reagent (Invitrogen), total RNA was isolated from 18 pairs of pterygium and normal conjunctival tissues (discovery phase *n* = 12; validation phase *n* = 6) from 18 pterygium patients. Twelve pairs of pterygium and normal conjunctival tissue RNA from the discovery phase were used independently for m6A and m6A modification-related regulators [include “Writer” (*METTL3*, *METTL14*, and *WTAP*), “Eraser” (*FTO* and *ALKBH5*), and “Reader” (*YTHDF1*)] expression level detection. For the remainder of 12 pairs of pterygium and normal conjunctival tissue RNA, every four equal independent RNA samples were pooled into two types of samples for MeRIP and RNA-seq analysis. Six pairs of pterygium and normal conjunctiva tissue RNA from the validation phase were used independently for hub gene validation. After quantifying the samples using a NanoDrop ND-1000 instrument, total RNA was reversed to cDNA using the Qiagen RNeasy kit according to manufacturer’s protocol (TaKaRa, 6110). Real-time qRT-PCR was performed on an ABI Prism 7500 Sequence Detection system using SYBR green reagent (Applied Biosystems); *GAPDH* was used as the endogenous control gene, the primers are listed in Supplementary Table 2. The relative gene expression levels were calculated using the 2-ΔΔCt method.

### Quantification of m6A in Total RNA

Total RNA from 18 pairs of pterygium and normal conjunctiva tissues from discovery phase was used for quantification of m6A modifications using an m6A RNA Methylation Quantification Kit (P-9005, EpiGentek). First, we prepared a negative control and also a positive control using six different concentrations of m6A ranging from 0.01 to 0.5 ng/μL, as recommended by the manufacturer. Then 2 μL positive control, 2 μL negative control, or 200 ng total RNA were bound in the 96-well plates. After removing the binding solution from each well, the samples were washed three times. The wells were incubated for 1 h with the addition of anti-m6a antibody and washed four times. Then, add the detection antibody to the wells, incubate at room temperature for 30 min and wash four times. Add the enhancer solution, incubate at room temperature for 30 min and then wash five times. Finally, add the developer solution and incubate at room temperature until the developer solution turns blue and then add the stop solution, the samples were analyzed on a SYNERGY 2 (BioTek) microplate reader at 450 nm; the absolute amount of m6A was quantified and the percentage of m6A in RNA was established as%m6A in total RNA.

### Immunohistochemistry Staining

Six pairs of pterygium and normal conjunctiva tissues from discovery phase were embedded in formalin. Each tissue was cut to 4-μm thick and mounted on a glass slide. Dewaxing sections were performed as previously described (Kawaguchi et al., 2002). Endogenous peroxidase activity was inhibited and blocked with 5% bovine serum albumin for 30 min at 37°C. The slices were incubated in anti-*METTL3* (Abcam, 1:400, ab195352) (Zhang et al., 2020b) overnight at 4°C, washed three times with PBS for 5 min, and then incubated with secondary anti-horseradish peroxide at 37°C for 30 min. After three more washes with PBS, the slices were visualized in diaminobenzidine chromogenic solution. Microscopic images were obtained by light microscopy (Carl Zeiss, Oberkochen, Germany).

### MeRIP Sequencing, RNA Sequencing, and Bioinformatics Analyses

m6A-modified RNA immunoprecipitation sequencing and RNA-seq were performed by Guangzhou Epibiotek Co., Ltd. Briefly, the Zymo RNA Clean and Concentrator-5 Kit was used to purify RNA from total RNA samples; then these were fragmented into 200 nucleotide lengths. A portion of the purified RNA was used for RNA-seq as input. The rest of the purified RNA was enriched via immunoprecipitation with an anti-m6A antibody (Abcam, cat. No. ab151230) in IP buffer (300 mM NaCl, 0.2% Igepal CA-630, 20 mM Tris-HCl, PH 7.4) overnight at 4°C. Then, the m6A-Ab mixture was incubated with protein-g magnetic beads (Thermo Fisher, cat. No. 88847) at 4°C for 2 h for immunoprecipitation. After washing and elution, IP RNA is obtained. Then, first-strand cDNA about m6A antibody-enriched RNAs and input RNAs were synthesized by SMART. The library of ultramicro-RNA methylated m6A and input RNAs were obtained by PCR amplification and library purification. Bioptic Qsep100 analyzer is used to conduct quality control over the library. Finally, the samples were sequenced using Illumina NovaSeq^TM^ 6000 in PE150 sequencing mode.

After obtaining clean data from Illumina NovaSeq^TM^ 6000, quality control was performed using Cutadapt (v2.5), including trimming aptamers and filtering sequences, and then the remaining reads were aligned to human pooled genome GRCh38 under parameters: “rna_strandness RF” using the Hisat2 aligner (v2.1.0). Mapped reads from the IP and input libraries were provided to the exomePeak R package (v2.13.2) to identify m6A peaks under the parameters: “PEAK_CUTOFF_PVALUE = 0.05, PEAK_CUTOFF_FDR = NA, FRAGMENT_LENGTH = 200.” Use the exomePeak R package to identify differential m6A peaks under the parameter: “PEAK_CUTOFF_PVALUE = 0.05, PEAK_CUTOFF_FDR = NA, FRAGMENT_LENGTH = 200.” Gene Ontology (GO) and Kyoto Encyclopedia of Genes and Genomes (KEGG) analysis were performed using clusterProfile R package (v3.6.0). m6A-RNA-related genomic features were visualized using Guitar R package (v1.16.0). Identified m6A peaks which *P* value <0.05 were chosen for the *de novo* motif analysis using homer (v4.10.4) under parameters: “−len 6 −rna.”

The expression of gene transcripts was quantified using featureCount (v1.6.3), and differentially expressed genes were identified by DESeq2 R package (1.18.1). Genes were considered as differentially expressed if gene expression is FC ≥ 2 and with a FDR ≤ 0.05.

All codes are available on GitHub (GitHub, Inc., San Francisco, CA, United States) at https://github.com/zxiahong/titre-trajectory-aje. To further explore the essential role of m6A mRNA modification in the development of pterygium, the identified m6A peaks and Differentially expressed genes (DEGs) were subjected to network analyses^1^ as previous described (Schuierer et al., 2010) (Figure 1B).

### Public Databases and Analysis

Expression profiling data (GSE83627, GSE51995, and GSE2513) were downloaded from the NCBI Gene Expression Omnibus (GEO)^2^ database. The expression levels of genes were analyzed using R software (version 4.0.2).^3^ The Student’s *t*-test was used to compare the relative gene expression between pterygium and normal conjunctiva tissues. GO and KEGG pathway analyses were performed based on significantly methylated genes and/or significantly expressed genes [|log2Fold Change (FC)| > 1 and *P*-value < 0.05] (Huang da et al., 2009).

## Results

### Levels of m6A and METTL3 Are Downregulated in Pterygium

**FIGURE 2:**
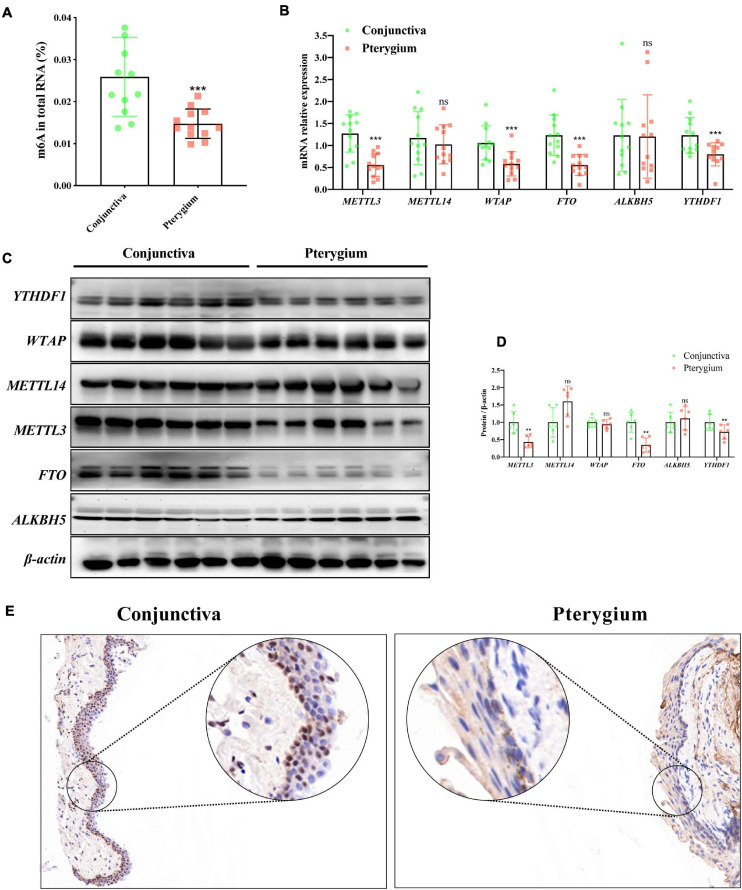
m6A and *METTL3* are downregulated in pterygium. **(A)** Quantification of m6A in total RNA in pterygium and conjunctiva tissues. *N* = 12 per group. **(B)** mRNA expression level of the major enzymes of m6A in pterygium and conjunctiva tissues: *METTL3*, *METTL14*, *WTAP*, *FTO*, *ALKBH5*, *YTHDF1*. *N* = 12 per group. **(C,D)** Protein expression levels of these enzymes determined via Western blotting **(C,D)** the results of corresponding densitometric analyses. *N* = 12 per group. **(E)** Immunohistochemistry staining of *METTL3* in pterygium and conjunctiva tissues. Scale bar: 25 μm. Results are expressed as the mean ± SEM (NS indicates not significant, **P < 0.01, ***P < 0.001, compared to the conjunctiva group).

First, we quantified m6A levels in pterygium and normal conjunctival tissues. Interestingly, as shown in Figure 2A, the level of m6A was significantly lower in pterygium than in normal conjunctival tissues (−0.01117 ± 0.00289, Fold-Change = 0.56892, *P*-value = 0.0008). Next, using qRT-PCR, we examined the mRNA expression levels of five crucial enzymes needed for m6A modification: *METTL3*, *METTL14*, *WTAP*, *FTO*, and *ALKBH5*. We found that the mRNA levels of *METTL3* were extraordinarily lower in pterygium versus conjunctive (−0.7155 ± 0.1434, Fold-Change = 0.43728, *P*-value < 0.0001, Figure 2B). A similar decrease in METTL3 was also found in Western blotting analyses (−0.2752 ± 0.08527, Fold-Change = 0.68424, *P*-value = 0.0091, Figures 2C,D) and immunohistochemistry staining (especially in epithelium, Figure 2E). Also, decreasing expression of FTO in mRNA and protein were also found qRT-PCR and western blotting. However, this trend violated the change of m6A modification. Taken together, these results indicate that *METTL3* is the key m6A modification regulator in pterygium.

### Overview of the m6A Methylation Map in Pterygium

**FIGURE 3:**
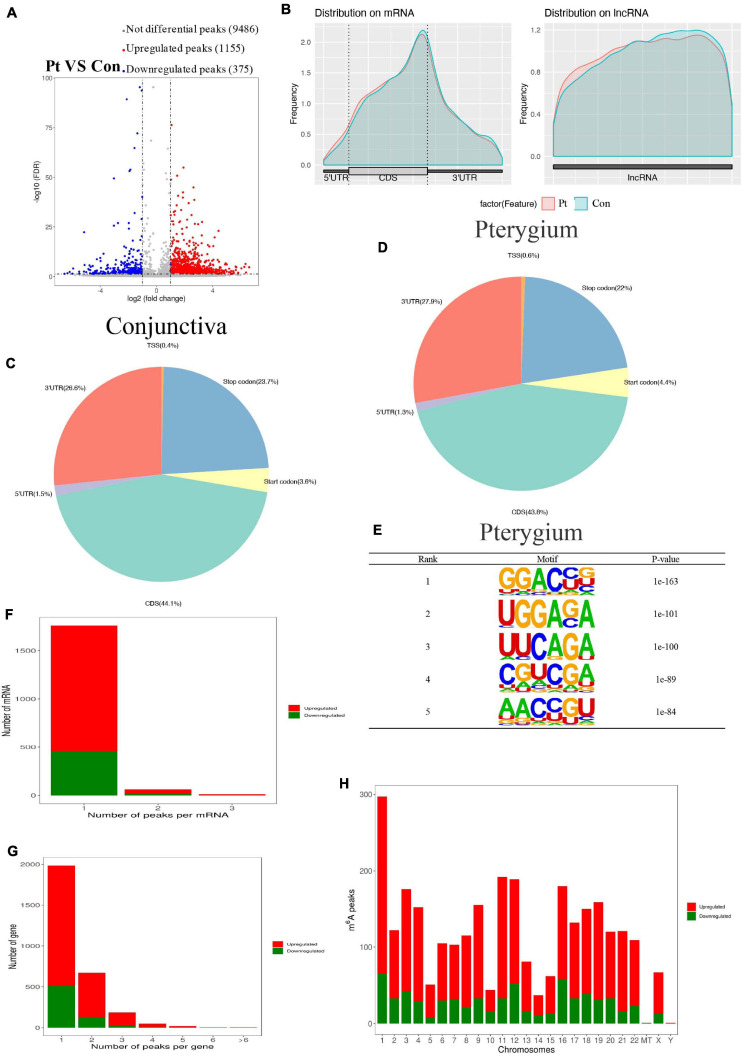
Overview of altered m6A-modified transcripts in pterygium. **(A)** Volcano plots showing significantly different m6A peaks. **(B)** Average m6A peaks in conjunctiva and pterygium. **(C,D)** Pie charts showing the distribution of m6A peaks in conjunctiva **(C)** and pterygium **(D)**. **(E)** Top five m6A motifs from the altered m6A peaks in pterygium. **(F)** Distribution of altered m6A peaks per mRNA. **(G)** Distribution of altered m6A peaks per gene. **(H)** Distribution of altered m6A peaks in human chromosomes. Fold change ≥2 and *P* < 0.05.

After pool four independent RNA from pterygium and normal conjunctiva to get two types of samples for the MeRIP sequence, we performed MeRIP-seq analysis on three pairs of pterygium and normal conjunctival samples. As shown in Figure 3A, compared to conjunctiva, pterygium had 1155 significantly upregulated m6A peaks and 375 downregulated m6A peaks (fold changes ≥ 2). Table 1 list the top 20 peaks, and Figure 3B shows the distribution of m6A signals around mRNA and lncRNA were comparable in the two classes of tissue samples. The tissues showed different patterns of peaks with a relative increase at the start codon region (4.4 vs. 3.6% for pterygium and conjunctiva, respectively) and in the 3′ untranslated region (3′UTR, 27.9 vs. 26.6%), and a relative decrease in the coding sequence (CDS, 43.8 vs. 44.1%) and at the stop codon (22 vs. 23.7%) (Figures 3C,D). Also, the classic GGAC motif (Fu et al., 2014; Liu et al., 2014) and the top five m6A motifs in pterygium (Figure 3E) and conjunctiva (Supplementary Figure 1A) were observed. Analyses of the distribution of m6A peaks indicated that most mRNAs and genes had m6A peaks (mRNAs with 458 downregulated peaks and 1,300 upregulated peaks; genes with 487 downregulated peaks and 1,368 upregulated peaks, Figures 3F,G). Variant peaks were found in all chromosomes, especially chr1, chr3, chr11, chr12, and chr16 (Figure 3H).

**TABLE 1:** Top 20 altered m6A peaks of m6A-modified mRNA in the pterygium vs conjunctiva.

Chromosome	Peak start	Peak end	Gene name	Regulation	Fold change	Peak region	*P* value
chr8	97144169	97144490	*AP003117.1*	Up	96.3357918	exon	1.0471E-06
chr20	38089777	38090167	*RPRD1B*	Up	22.7848031	3′UTR	8.3176E-07
chr7	152181603	152181904	*KMT2C*	Up	13.3614067	3′UTR	1.7783E-07
chr2	67402830	67402950	*ETAA1*	Up	12.5533456	CDS	1.2589E-18
chr1	25498324	25498714	*TMEM57*	Up	9.71355908	3′UTR	3.8905E-07
chr6	84371809	84372380	*FAM83B*	Up	9.31786869	CDS	1.5849E-23
chr10	54870279	54870669	*CCSER2*	Up	9.31786869	CDS	2.5119E-13
chr1	39485587	39485858	*MACF1*	Up	6.96440451	CDS	2.5119E-17
chr22	41436593	41436773	*TOB2*	Up	6.63455637	exon	1.9953E-13
chr1	186420419	186420749	*C1orf27*	Up	6.58872814	3′UTR	5.4954E-08
chr10	122514191	122514729	*HTRA1*	Down	0.32308821	3′UTR	3.9811E-16
chr17	80394078	80394439	*RNF213*	Down	0.31425334	3′UTR	1.0965E-05
chr7	94430211	94430392	*COL1A2*	Down	0.26061644	exon	7.9433E-14
chr8	56963672	56964159	*IMPAD1*	Down	0.25881623	CDS	1.2589E-11
chr4	123401555	123402271	*SPRY1*	Down	0.25525303	CDS	6.3096E-30
chr7	116699611	116700211	*MET*	Down	0.25525303	CDS	7.9433E-12
chr1	40240113	40240503	*RLF*	Down	0.25	CDS	3.6308E-08
chr7	100036589	100036770	*ZKSCAN1*	Down	0.2381595	3′UTR	2.5704E-10
chr14	76202383	76202534	*GPATCH2L*	Down	0.13584186	3′UTR	1.2882E-10
chr15	64300481	64300722	*AC087632.1*	Down	0.06470406	5′UTR	2.3988E-06

*3′UTR, 3′ untranslated region; 5′UTR, 5′ untranslated region; CDS, coding sequence; exon, expressed region.*

### Differentially Methylated mRNAs Participate in Important Signaling Pathways

**FIGURE 4:**
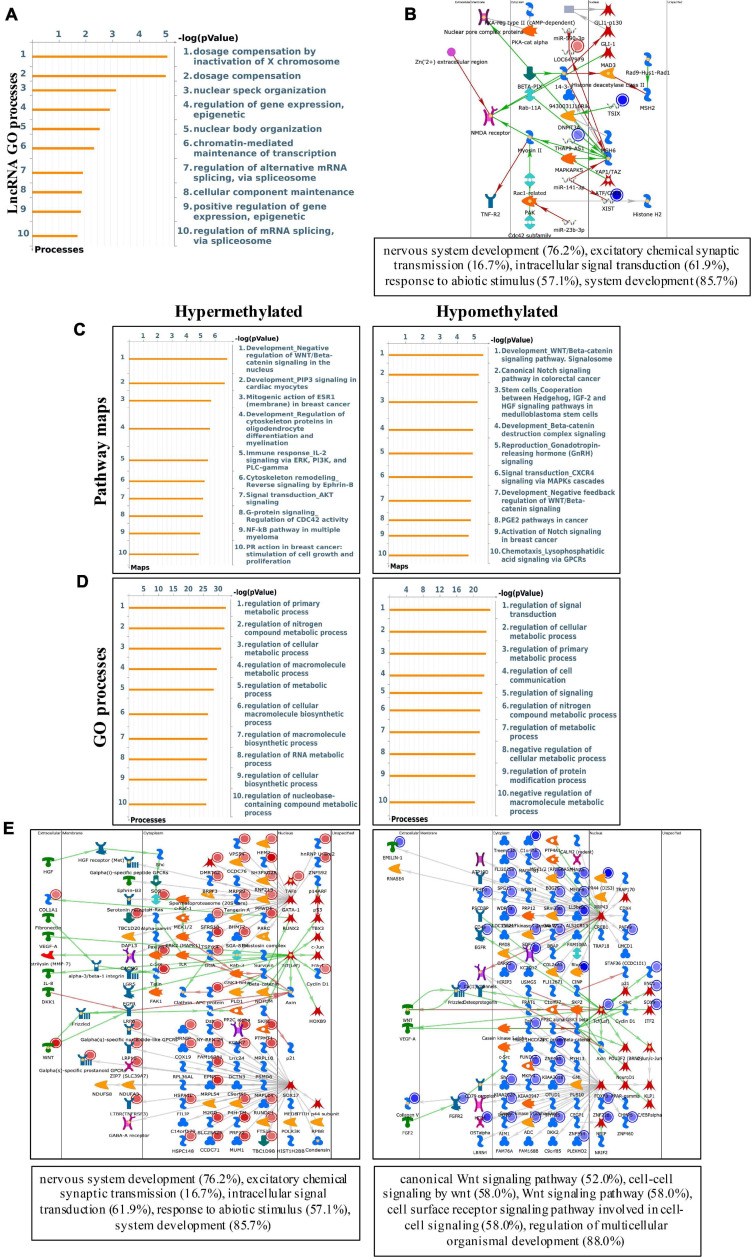
Systematical analyses of differentially methylated mRNA in pterygium. **(A)** Gene Ontology (GO) processes analysis of differentially methylated lncRNA. **(B)** Top three scored networks analysis of differentially methylated lncRNA. **(C)** Pathway maps analysis of differentially methylated peaks. Left panel represents hypermethylated genes and right panel represents hypomethylated peaks. **(D)** GO process analysis of differentially methylated peaks. Left panel represents hypermethylated peaks and right panel represents hypomethylated peaks. **(E)** Top three scored networks analysis of differentially methylated peaks. Left panel represents hypermethylated genes and right panel represents hypomethylated peaks.

**FIGURE 5:**
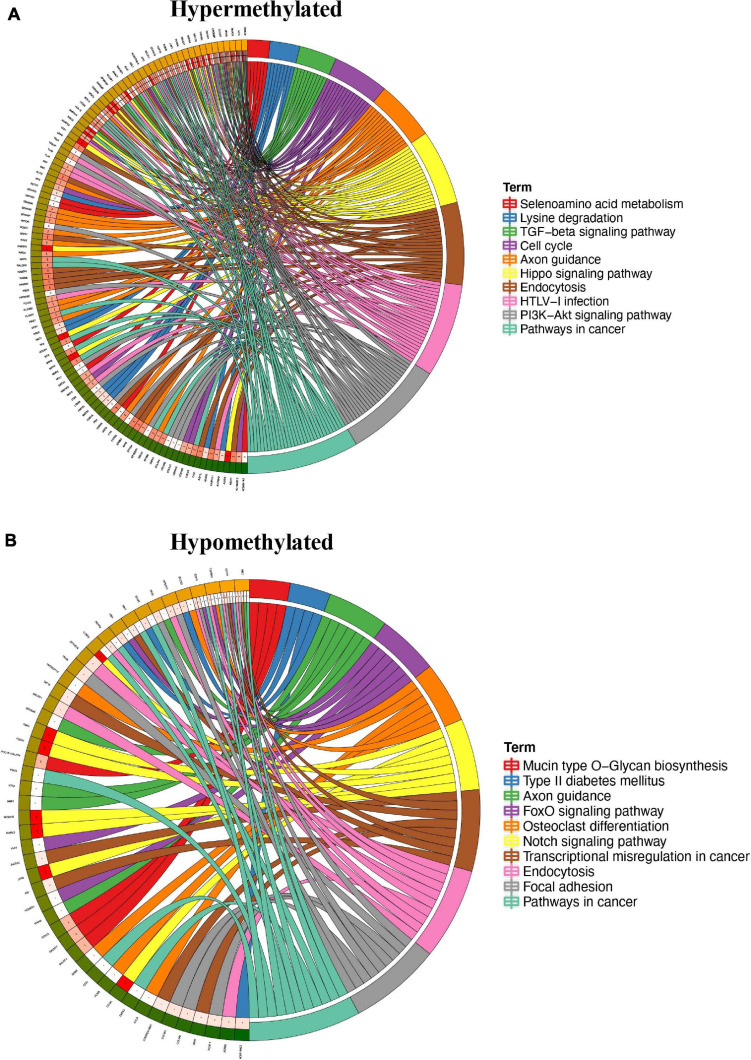
KEGG enrichment analyses of differentially methylated mRNA in pterygium. Top 10 KEGG pathways of genes with **(A)** upregulated m6A peaks and **(B)** downregulated m6A peaks. Squares in left semicircle refer to the upregulated or downregulated mRNAs, the squares in right semicircle refer to the pathways. *P < 0.05, **P < 0.01, ***P < 0.001, genes compared to pathways.

To study the biological significance of m6A modification in pterygium, GO and KEGG pathway analyses of differentially methylated mRNAs were conducted. For GO analyses, we considered biological processes (BP), cellular components (CC), and molecular functions (MF). The main results are given in Supplementary Figure 1B. Using Metacore system, we tested dys-methylated lncRNA which were significantly enriched in dosage compensation by inactivation of X chromosome, dosage compensation and nuclear speck organization (Figure 4A). In addition, the top scored network analyses of these were found to be relative with nervous system development (76.2%), excitatory chemical synaptic transmission (16.7%), intracellular signal transduction (61.9%), response to abiotic stimulus (57.1%), system development (85.7%) (Figure 4B and Supplementary Table 3). In the pathway map analysis of mRNA, the hypermethylated peaks were strongly associated with negative regulation of WNT/Beta-catenin signaling in the nucleus. Besides, the most relevant biological pathways of hypomethylated peaks were WNT/Beta-catenin signaling pathway (Signalosome) and Canonical Notch signaling pathway in colorectal cancer (Figure 4B). In addition, GO process results showed that the hypermethylated peaks were significantly enriched in signal transduction (ESR1-nuclear pathway) and cell adhesion (Cadherins), while the hypomethylated peaks were enriched in regulation of signal transduction and regulation of cellular metabolic process (Figure 4C). Furthermore, the top three scored network analyses of hypermethylated peaks about the development of pterygium were found to be involved in nervous system development (76.2%), excitatory chemical synaptic transmission (16.7%), intracellular signal transduction (61.9%), response to abiotic stimulus (57.1%), system development (85.7%) (Figure 4D and Supplementary Table 3). The top three scored networks of hypomethylated peaks were enriched in canonical Wnt signaling pathway (52.0%), cell-cell signaling by wnt (58.0%), Wnt signaling pathway (58.0%), cell surface receptor signaling pathway involved in cell-cell signaling (58.0%), regulation of multicellular organismal development (88.0%) (Figure 4E and Supplementary Table 3). Additionally, in KEGG pathway analyses, the hippo signaling pathway, endocytosis, and cancer-related pathways were significantly correlated with genes that showed upregulated m6A peaks in pterygium (Figure 5A). Genes with downregulated m6A peaks were significantly correlated with the Notch signaling pathway and Mucin type O-Glycan biosynthesis (Figure 5B).

### Overview of Transcriptome Profiles and Conjoint Analyses of MeRIP-Seq and RNA-Seq Data

**FIGURE 6:**
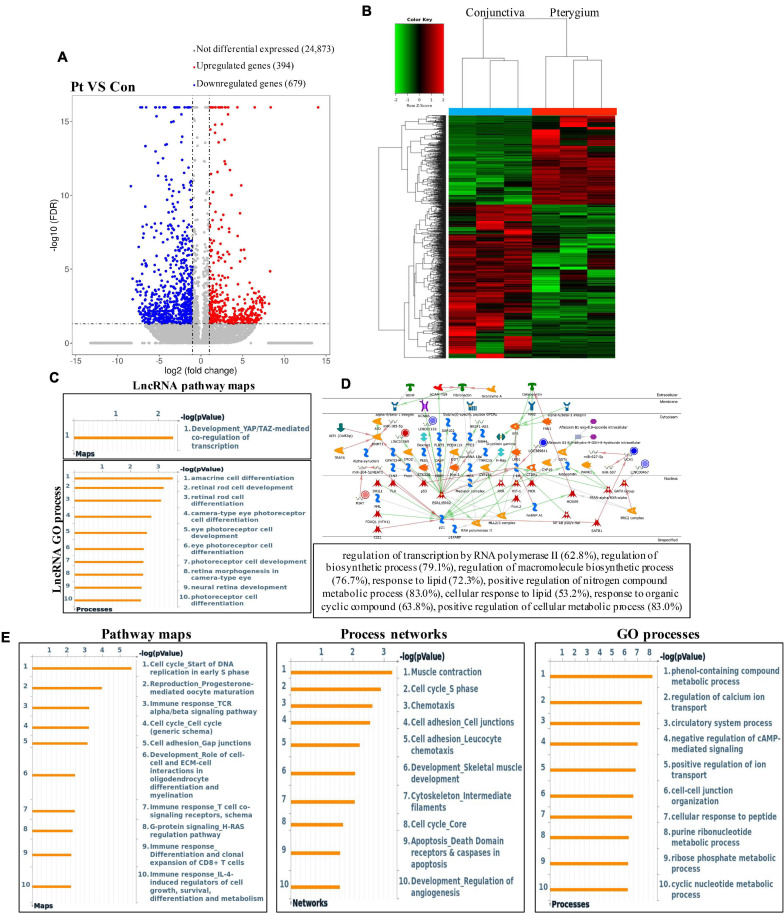
Results of significant RNA of pterygium in RNA-seq data. **(A)** Volcano plots and **(B)** heatmap plots showing differentially expressed genes in pterygium vs. conjunctiva. Fold change ≥2 and *P* < 0.05. **(C)** GO processes and pathway maps analysis of significant lncRNA. **(D)** Top three scored networks analysis of significant lncRNA. **(E)** Pathway maps, process networks and GO processes analysis of significant mRNA. Left panel represents pathway maps, middle panel represents process networks and right panel represents GO processes.

First, we tested the transcriptome profiles of altered genes in three pairs of pterygium and normal conjunctival samples using RNA-seq. Compared to normal conjunctival samples, pterygium samples had 394 significantly upregulated genes and 679 significantly downregulated genes (Figures 6A,B). The top 20 differentially expressed genes are listed in Table 2. After the systematically analyzed by Metacore, we found the different expression of lncRNA were significantly enriched in development (*YAP/TAZ* mediated co regulation of transcription) in pathway maps and serval types of cells differentiation in GO processes (Figure 6C). in addition, the top three scored network analyses of these were found to be strongly associated with regulation of transcription by RNA polymerase II (62.8%), regulation of biosynthetic process (79.1%), regulation of macromolecule biosynthetic process (76.7%), response to lipid (72.3%), positive regulation of nitrogen compound metabolic process (83.0%), cellular response to lipid (53.2%), response to organic cyclic compound (63.8%), positive regulation of cellular metabolic process (83.0%) (Figure 6D and Supplementary Table 4). Additionally, cell cycle (Start of DNA replication in early S phase) in the pathway maps analysis, muscle contraction in process networks and phenol-containing compound metabolic process in GO processed were the top three enrichment in different expression of mRNA (Figure 6E).

**TABLE 2:** Top 20 differentially expressed genes in pterygium vs conjunctiva after RNA-seq.

Gene name	Chromosome	Location	Regulation	Log2 (FC)	FDR
*MPZ*	1	161303594.161309969	Down	−8.4045665	2.4E-11
*CCDC144A*	17	16666571.16787421	Down	−8.1943654	0.0000441
*C2CD4A*	15	62066977.62070917	Down	−8.175038	0.001090807
*NRAV*	12	120490338.120495946	Down	−7.7763482	2.41E-04
*ZNF784*	19	55620741.55624566	Down	−7.6855849	6.80E-05
*HNRNPA1P16*	17	2306762.2308048	Down	−7.6074514	3.38E-04
*OR5P1P*	11	7772893.7773814	Down	−7.5687836	1.17E-04
*AC073111.5*	7	150410557.150410887	Down	−7.4599851	3.08E-07
*AC136475.1*	11	318640.325631	Down	−7.4390189	7.08E-03
*AC018529.2*	18	77112602.77115726	Down	−7.4053031	4.37E-03
*PPBP*	4	73986439.73988190	Up	7.37564673	0.003693999
*AC243829.1*	17	36183235.36196471	Up	7.38059416	1.29E-03
*DPYSL4*	10	132186948.132205759	Up	7.60525389	0.000776864
*GPR1*	2	206175316.206218047	Up	7.65852146	0.001892264
*ATP6V0A4*	7	138706294.138799560	Up	7.68141129	9.71E-03
*CXCR1*	2	218162841.218166962	Up	8.16943888	2.16E-03
*AC092111.1*	12	8217758.8221115	Up	8.2987707	1.40E-05
*CLDN5*	22	19523024.19527545	Up	8.36106543	0
*CRNN*	1	152409243.152414263	Up	14.0548696	0.00E + 00
*TMPRSS11B*	4	68226653.68245694	Up	14.0631053	0.00E + 00

**FIGURE 7:**
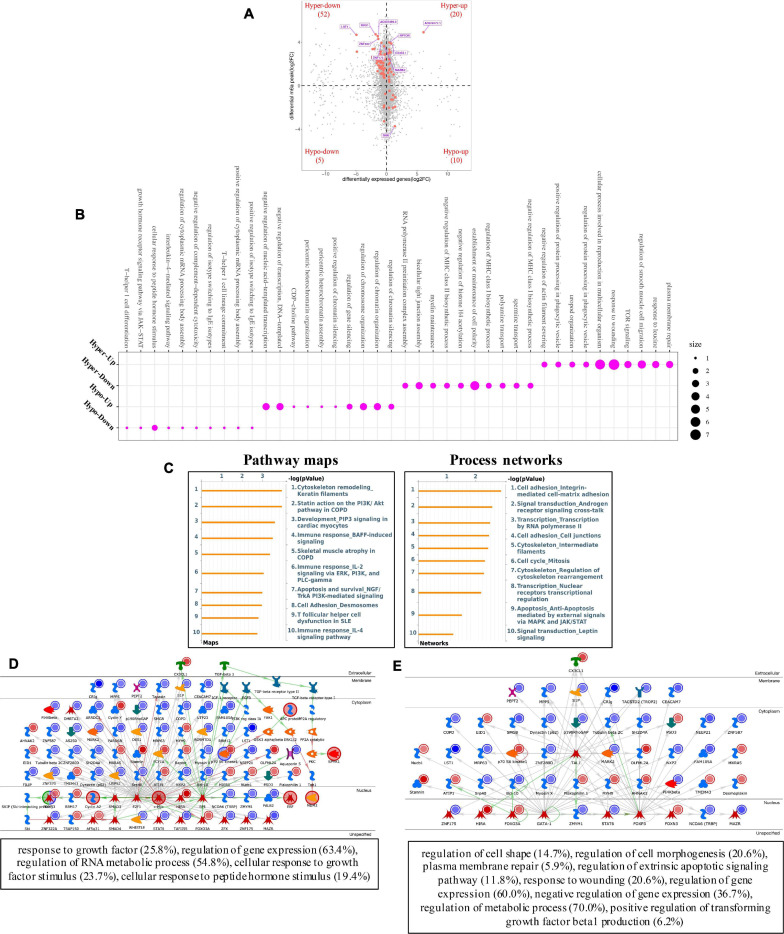
Systematical analyses of MeRIP-seq and RNA-seq data in pterygium. **(A)** Significantly m6A-modified RNA identified after conjoint analyses of MeRIP-seq and RNA-seq data. **(B)** Bubble diagram of GO biological process categories enriched for DEGs with m6A hyper- or hypo-methylated. **(C)** Pathway maps analysis **(left)** and Process networks analysis **(right)** of differentially m6A-modifed genes. **(D)** Top three scored networks analysis of m6A-modified genes. **(E)** Key transcription factors and target genes network analysis of m6A-modifed genes. DEGs, differentially expressed genes.

Furthermore, conjoint analyses of MeRIP-seq and RNA-seq data were performed (Figure 7A). We identified 72 hypermethylated m6A peaks in mRNAs that were significantly upregulated (20) or downregulated (52), while 15 hypomethylated m6A peaks in mRNAs were significantly upregulated (10) or downregulated (5). Next, 87 genes that showed significant changes in both m6A modification and RNA expression levels were subjected to GO, pathway analyses and networks analyses. The GO analyses of processes associated with the four gene sets showed detail in Figure 7B, which apparently linked numerous functional processed and pathways. Interestingly, we found Cytoskeleton remodeling (Keratin filaments) and Statin action on the *PI3K/Akt* pathway in COPD were enriched in pathway maps, and Cell adhesion (Integrin-mediated cell-matrix adhesion) and Signal transduction (Androgen receptor signaling cross-talk) were enriched in process networks (Figure 7C). In addition, the top 20 GO and KEGG pathways that were up- or downregulated in both m6A modification and RNA expression genes are shown in Supplementary Figures 1C–E. Moreover, the top three scored network analyses indicated that m6A-modified genes of pterygium were significantly enriched in response to growth factor (25.8%), regulation of gene expression (63.4%), regulation of RNA metabolic process (54.8%), cellular response to growth factor stimulus (23.7%), cellular response to peptide hormone stimulus (19.4%) (Figure 7D and Supplementary Table 5). After analyzed the key transcription factors and target genes in m6A-modified genes, we showed the top three transcription factors (*GATA-1*, *FOXP3*, and *TAL1*) and the m6A-modified genes which most enriched in regulation of cell shape (14.7%), regulation of cell morphogenesis (20.6%), plasma membrane repair (5.9%), regulation of extrinsic apoptotic signaling pathway (11.8%), response to wounding (20.6%), regulation of gene expression (60.0%), negative regulation of gene expression (36.7%), regulation of metabolic process (70.0%), positive regulation of transforming growth factor beta1 production (6.2%) (Figure 7E). These observations indicate that genes with m6A modification may play important role in the development of pterygium.

### Validating the 87 Genes That Showed Significant Changes in Both m6A Modification and RNA Expression Levels Against the GEO Datasets

**FIGURE 8:**
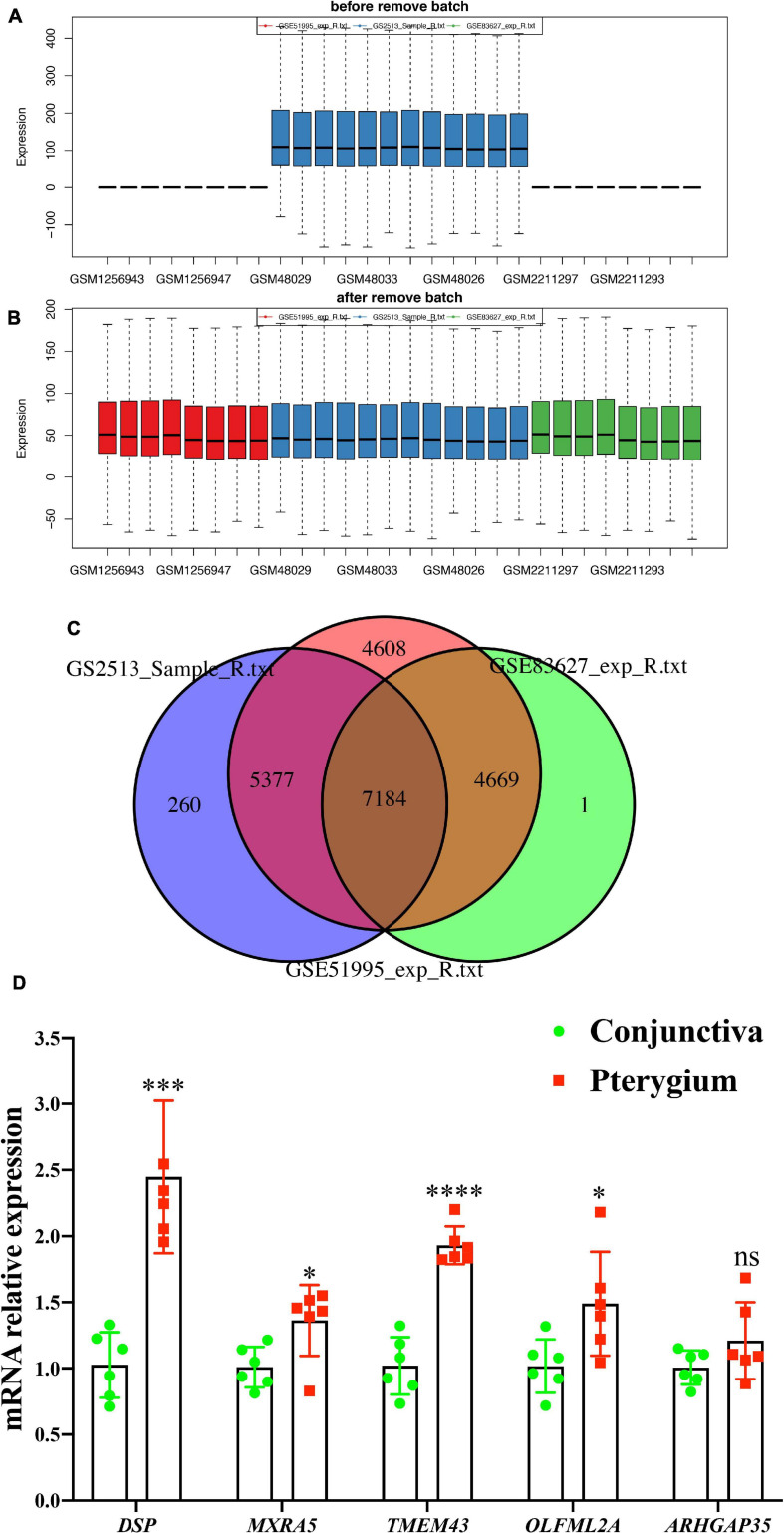
Validation gene expression in GEO datasets. **(A,B)** The expression of genes from GEO datasets (GSE2513, GSE51995, and GSE83627) were normalized to remove batch effects. **(C)** Wayne chart of the three GEO datasets. **(D)** mRNA expression level of five hub genes (*DSP*, *MXRA5*, *TMEM43*, *OLFML2A*, and *ARHGAP35*) determined via qPCR. *N* = 6 per group. Results are expressed as the mean ± SEM (NS indicates not significant, *P < 0.05, **P < 0.01, ***P < 0.001, ****P < 0.0001 compared to the conjunctiva group).

To further validate the most relevant gene correlations with pterygium, we examined 87 genes that showed significant changes in both m6A modification and RNA expression levels in GEO datasets including GSE83627, GSE51995 (Hou et al., 2014), and GSE2513 (Wong et al., 2006) (Figures 8A–C). Finally, five hub genes showed consistent difference in three GEO datasets and our study. Their expression levels in each tissue were validated by qRT-PCR (Figure 8D). The expressions of *DSP*, *MXRA5*, *TMEM43*, and *OLFML2A* were increased, similar to our sequence data, while the decrease in expression of *ARHGAP35* was not found. The patterns of m6A modification in these five genes are visualized Supplementary Figure 2.

## Discussion

Epigenetic modification is associated with many diseases (Chen et al., 2017; Rezasoltani et al., 2017). RNA modifications, especially m6A, were reported as a novel territory in epigenetic modification to be much associated with various diseases nowadays, such as tumorigenesis, cardiovascular diseases, inflammation, and Type II diabetes (He et al., 2013; Gustavsson et al., 2014; Rezasoltani et al., 2017; Dorn et al., 2019; Zhang L. et al., 2020). However, the role of m6A modification in pterygium development has not yet been elucidated.

In this study, we provide the first data analysis in pterygium. We found significantly downregulated m6A levels in pterygium compared to conjunctival tissues, especially by *METTL3*, which may be the pivotal regulator in pterygium. Interestingly, *METTL3* expression has been found to play a role in the regulation of cell growth-related pathways as well as cell death-related pathways in cancer (Barbieri et al., 2017; Chen et al., 2018). And, previous studies showed the cell growth-related pathways and cell death-related pathways were important for the development of pterygium.

Further analyses identified significantly up- and downregulated m6A peaks, the potential functions of altered m6A-modified transcripts, and synchronously differentially expressed genes that showed both m6A modification and RNA expression. Overall, the analysis shows an altered landscape of m6A modification is pterygium.

In our MeRIP-seq analysis, we found 458 downregulated m6A peaks and 1,300 upregulated m6A peaks on mRNA. We conducted GO and KEGG analysis to study the biological functions of these methylated mRNAs. We found dys-methylated lncRNA were significantly enriched in dosage compensation by inactivation of X chromosome. Dosage compensation of the X chromosome is the inactivation of one of the two X chromosomes by heterochromatization so that only one chromosome is active, resulting in a balanced dose of gene products on the X chromosome in males and females, although the number of X chromosomes is not the same (Jordan et al., 2019). Dosage compensation of the X chromosome is associated with epigenetics as well as gene expression patterns (Brockdorff and Turner, 2015), and long non-coding RNAs are key players in the regulation of the X chromosome (Sahakyan et al., 2018). Therefore, aberrantly methylated lncRNAs may cause alterations in the epigenetics of pterygium as well as gene expression patterns by regulating the inactivation of the X chromosome.

In KEGG pathway analyses, we found that upregulated m6A modification genes are related to the Hippo signaling pathway, which was first discovered in *Drosophila* and is a highly conserved kinase cascade composed of tumor suppressor Hippo (*MST* and *Lats* in mammals) and the downstream effector molecule *Yki* (*YAP* and *TAZ* in mammals). The latter is a transcriptional co-activator of target genes involved in cell proliferation and survival (Pan, 2010). Many studies have shown that the Hippo signal transduction pathway can regulate the transcriptional co-activation molecules *YAP* and *TAZ* through negative regulation, thereby regulating organ size, cancer occurrence, tissue regeneration, and stem cell function (Mo et al., 2014; Yu et al., 2015). *YAP* and *TAZ* are transcriptional co-activators that shuttle between the cytoplasm and the nucleus. They recognize homologous cis-regulatory elements by interacting with other transcription factors such as *TEA* domain family members, which are involved in cell proliferation, tissue regeneration, and stem cell maintenance (Piccolo et al., 2014; Chen M. et al., 2020). The continuous activation of this system can promote abnormal cell proliferation (Koo and Guan, 2018). *YAP* can regulate the cell cycle by controlling the cyclin to enter the S phase or inducing other proto-carcinoma transcription factors such as *c-Myc* (Zanconato et al., 2015). The occurrence of pterygium is related to cell proliferation, cell cycle changes, and a lack of limbal stem cells. The Hippo signal transduction pathway is most relevant to pterygium, suggesting that m6A modification may promote the occurrence and development of pterygium by regulating the Hippo signal transduction pathway to affect cell proliferation, cell cycle changes, and loss of limbal stem cell function. We also found that downregulated m6A modification genes were related to the Notch signaling pathway, which is involved in regulating various cellular processes of normal development, such as cell proliferation, differentiation, the epithelial–mesenchymal transition, angiogenesis, and many biological processes that promote the occurrence and development of cancer (Aster et al., 2017). The *Notch* pathway is mainly composed of four parts: *Notch* receptor (*Notch1–4*), Notch ligand (*Delta-like [Dll] 1*, *Dll3*, *Dll4*, *Jagged-1*, and *Jagged-2*), CSL-DNA binding protein, and downstream target genes (Meurette and Mehlen, 2018). Zhang et al. (2017) showed that *Notch4* is significantly highly expressed in human prostate cancer (PCa), and can promote the progression and metastasis of PCa through cell growth, apoptosis, migration, invasion, and the EMT. Another study (De Francesco et al., 2018) showed that *Notch*-related signaling pathways can promote the occurrence and development of breast cancer by regulating the EMT. However, the precise functions of this pathway in pterygium remain to be discovered and the m6A-modified genes related to this pathway in pterygium must be further elucidated.

Combining MeRIP-seq and RNA-seq data, we identified 87 differentially methylated m6A peaks and synchronously differentially expressed in mRNA. The expression of these genes may be regulated by m6A modification. Using GEO databases, we also identified five hub genes (*DSP*, *MXRA5*, *ARHGAP35*, *TMEM43*, and *OLFML2A*) that were most correlated with the development of pterygium. *DSP* encodes the desmoplakin protein, and is a critical component of desmosome structures in cardiac muscle and epidermal cells (Arnemann et al., 1991; Bornslaeger et al., 1996). Other studies have reported different roles of *DSP* in cancer (Papagerakis et al., 2009; Yang et al., 2012), but it is generally clear that it is a key factor in the EMT. *BMP6* significantly upregulates the expression of desmoplakin and desmoglein in human corneal limbal epithelial cells, consistent with its pro-differentiation role (Tiwari et al., 2020). Matrix-remodeling associated 5 protein, which is encoded by *MXRA5*, is a novel biomarker in colorectal cancer (Wang et al., 2013) and non-small-cell lung carcinoma (Xiong et al., 2012). The high expression of *MXRA5* is strongly associated with the remodeling of the extracellular matrix (ECM). *MXRA5* expression may also be involved in *TGF*β activation, which plays an important role in several developing connective tissues (Robertson et al., 2015; Bertoni et al., 2016). Interestingly, the high expression of *MXRA5* in our study suggests that it may remodel the ECM by activating *TGF*β in pterygium. Transmembrane protein 43 (*TMEM43*) plays an important role in maintaining the structure of the nuclear envelope (Wiemann et al., 2001), and mutations in *TMEM43* are strongly associated with arrhythmogenic right ventricular cardiomyopathy (Baskin et al., 2013). The high expression of *TMEM43* in pterygium, as in cancer, may act as a critical component in the *EGFR* signaling network to control cell survival, migration, and invasion (Jiang et al., 2017). The *ARHGAP35* gene encodes glucocorticoid receptor DNA binding factor-1 (also known as *p190RHOGAP*), which belongs to the large G protein/GTPase activation protein family. It can regulate rho-dependent signal transduction processes, such as cell adhesion, migration, invasion, and cytokinesis (Héraud et al., 2019). Its downregulation promotes the transformation and growth of epithelial tumor cells (Sun et al., 2014). In pterygium, it increases cell invasion and migration, thereby inducing or promoting the development of pterygium. *OLFML2A* is a novel regulator of transforming growth factor-β-induced smooth muscle differentiation and a novel Carcinogenic factor in liver hepatocellular carcinoma (Shi et al., 2014; Bai et al., 2020). Its role in pterygium has not been illustrated and further research is required.

The m6A modification plays an important role in the regulation of gene expression, and an abnormal level of m6A modification may be related to human diseases or cancer. It has been found that enzymes related to m6A modification may affect the UV-induced DNA damage response (Lin et al., 2016), tumor formation (Cui et al., 2017), and cell development and differentiation (Li et al., 2017). The decrease in m6A modification level caused by the downregulation of *METTL3* may be an important reason for the development of pterygium, but further research is needed to clarify the exact mechanism. Our research has identified many genes that are hypermethylated or hypomethylated and differentially expressed simultaneously. These genes may play an important role in the development of pterygium. Our findings suggest that adjusting the level of m6A modification may become a new treatment strategy for pterygium in the future.

## Conclusion

High-throughput sequencing produced a broad map of the m6A transcriptome in pterygium. We conducted joint analyses of MeRIP-seq and RNA-seq data and identified many differentially expresses m6A methylation peaks and differentially expressed genes, indicating that there is a potential connection between m6A methylation and gene expression. In addition, the genes we selected are related to the occurrence of pterygium.

## Data Availability Statement

The datasets presented in this study can be found in online repositories. The names of the repository/repositories and accession number(s) can be found below: NCBI GEO; GSE167933.

## Ethics Statement

This study was performed in accordance with the principles of the Declaration of Helsinki, and the study protocol was reviewed and approved by the Ethics Committee of Yangpu Hospital. Informed consent was provided by all subjects enrolled in this study. The ethical approval number is LL-2018-WSJ-010. The patients/participants provided their written informed consent to participate in this study.

## Author Contributions

YC and CY designed the work and prepared the manuscript. YJ collected the samples. YJ, XZ, and XYZ performed the experiments, conducted data analyses, and wrote the manuscript. KZ and JZ analyzed and interpreted the data. All authors discussed the results, and read and approved the final version of the manuscript for publication.

## Conflict of Interest

The authors declare that the research was conducted in the absence of any commercial or financial relationships that could be construed as a potential conflict of interest.

## Footnotes

1 https://portal.genego.com

2 https://www.ncbi.nlm.nih.gov/gds/

3 www.r-project.org

## Supplementary Material

Supplementary Figure 1 Serval enrichment analyses of pterygium. **(A)** Top five m6A motifs from the altered m6A peaks in conjunctiva. **(B)** Top 15 BP, MF, and CC GO terms of genes with **(up panel)** upregulated m6A peaks and **(down panel)** downregulated peaks. **(C,D)** Top 20 **(C)** GO enrichment and **(D)** KEGG enrichment terms of genes with significant changes in both m6A modification and mRNA levels. GO, Gene Ontology; BP, biological processes; CC, cellular components; MF, molecular functions.

Supplementary Figure 2 Results of visualization analyses of five hub genes. **(A–E)** The m6A level and expression of five hub genes in each tissue type were visualized using Integrative Genomics Viewer.

Supplementary Table 1 The detail of clinical information about 24 pterygium patients.

Supplementary Table 2 List of primers used for qRT-PCR.

Supplementary Table 3 Most relevant networks of lncRNA and hyper- or hypo-methylated peaks.

Supplementary Table 4 Most relevant networks of lncRNA from RNA-seq.

Supplementary Table 5 Most relevant networks of m6A-modified genes.
