# Supplementary figures and images for: Comprehensive Analysis of the Transcriptome-Wide m6A Methylome in Pterygium by MeRIP Sequencing
Source: Front Cell Dev Biol. 2021 Jun 25;9:670528. doi: 10.3389/fcell.2021.670528 (PMC8267473; doi:10.3389/fcell.2021.670528)

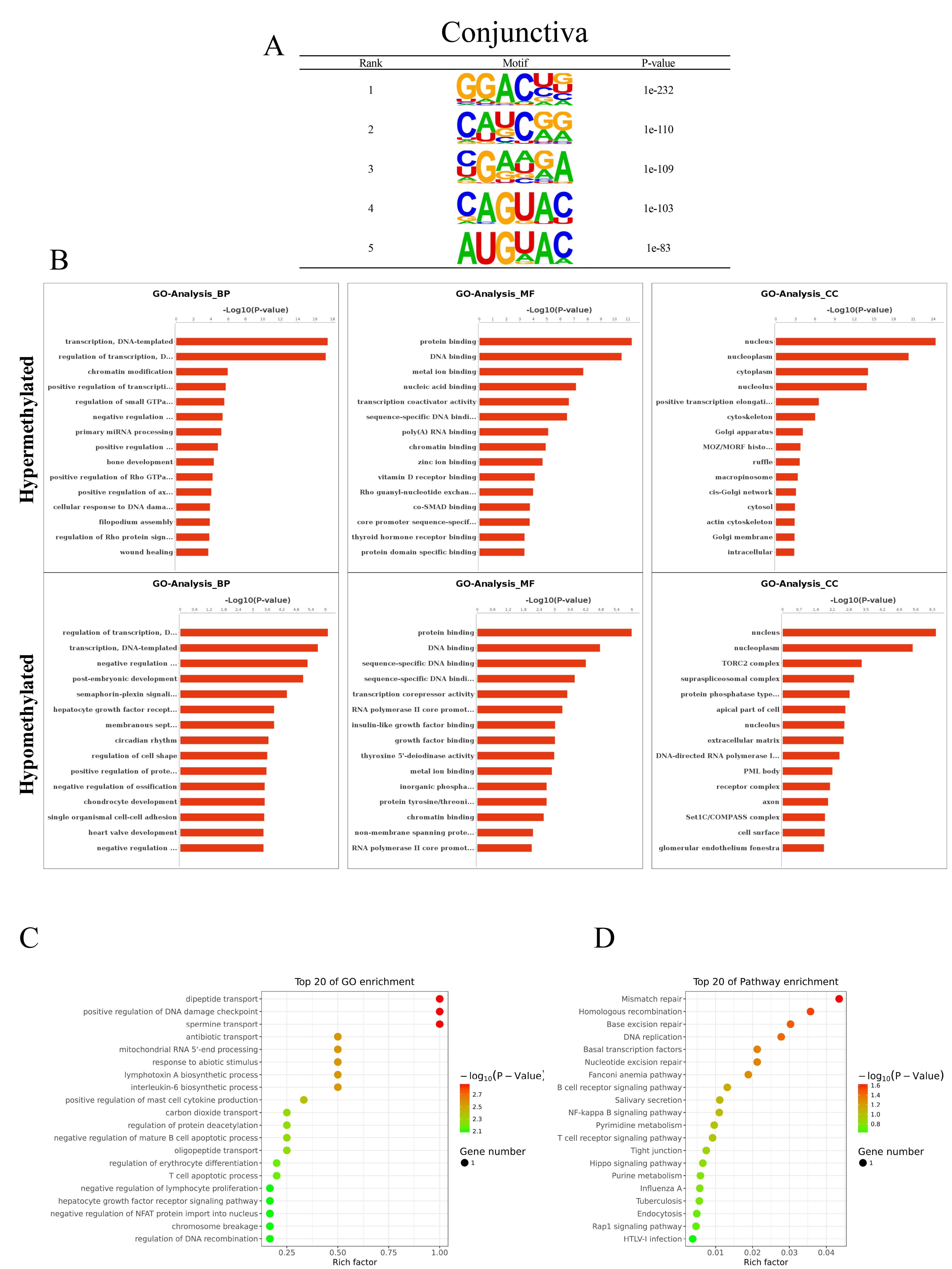

Supplement: Supplementary Figure 1 — Serval enrichment analyses of pterygium. (A) Top five m6A motifs from the altered m6A peaks in conjunctiva. (B) Top 15 BP, MF, and CC GO terms of genes with (up panel) upregulated m6A peaks and (down panel) downregulated peaks. (C,D) Top 20 (C) GO enrichment and (D) KEGG enrichment terms of genes with significant changes in both m6A modification and mRNA levels. GO, Gene Ontology; BP, biological processes; CC, cellular components; MF, molecular functions. [file Image_1.JPEG]

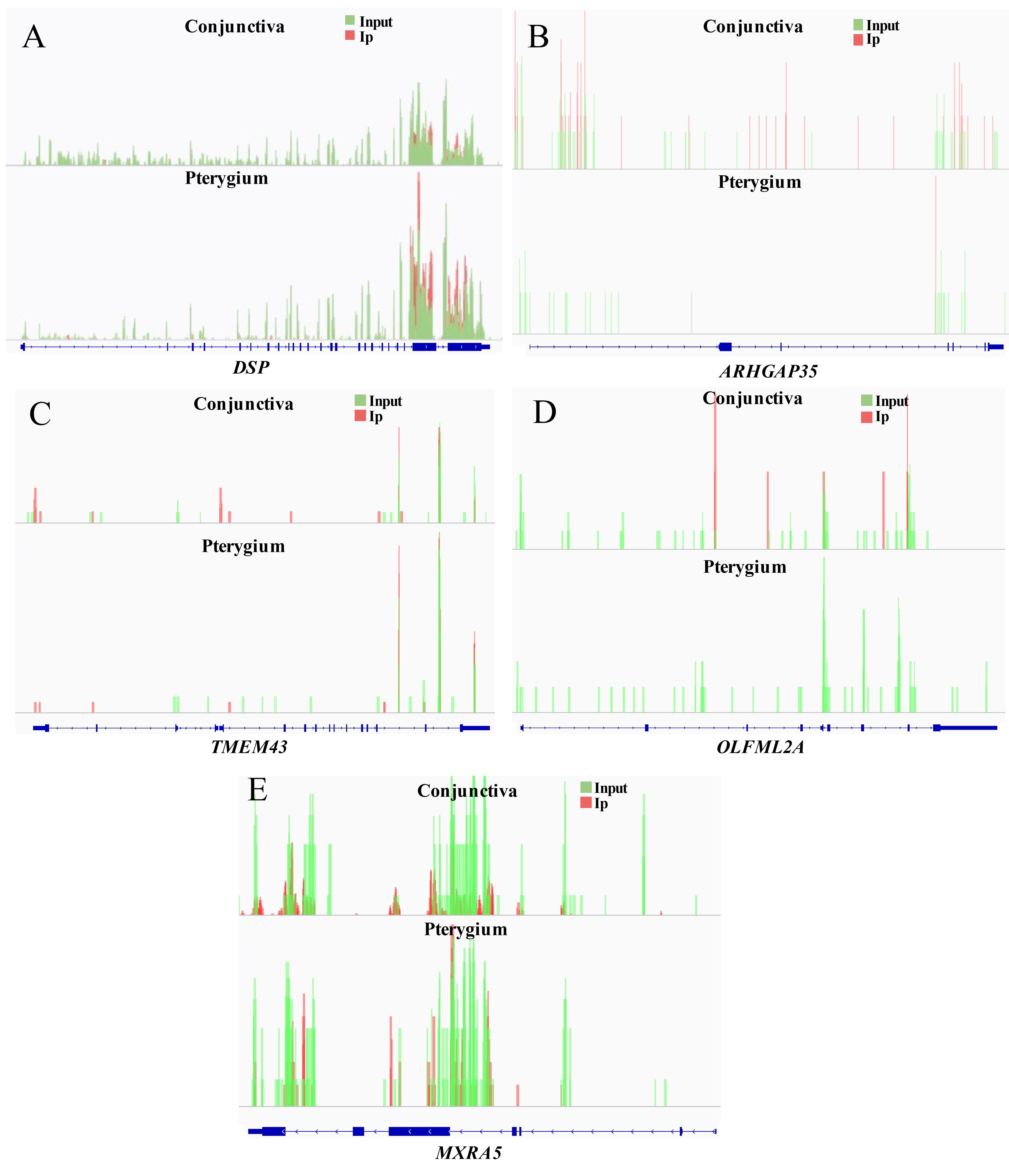

Supplement: Supplementary Figure 2 — Results of visualization analyses of five hub genes. (A–E) The m6A level and expression of five hub genes in each tissue type were visualized using Integrative Genomics Viewer. [file Image_2.JPEG]
